# Differences in Alzheimer’s Disease and Related Dementias Pathology Among African American and Hispanic Women: A Qualitative Literature Review of Biomarker Studies

**DOI:** 10.3389/fnsys.2021.685957

**Published:** 2021-07-21

**Authors:** Sarah K. Royse, Ann D. Cohen, Beth E. Snitz, Caterina Rosano

**Affiliations:** ^1^Department of Epidemiology, Graduate School of Public Health, University of Pittsburgh, Pittsburgh, PA, United States; ^2^Department of Psychiatry, University of Pittsburgh School of Medicine, Pittsburgh, PA, United States; ^3^Department of Neurology, University of Pittsburgh School of Medicine, Pittsburgh, PA, United States

**Keywords:** Alzheimer’s disease, African American, Hispanic, sex differences, tau, neurodegeneration, cerebral small vessel disease, amyloid

## Abstract

**Introduction:**

The population of older adults with Alzheimer’s disease and Related Dementias (ADRD) is growing larger and more diverse. Prevalence of ADRD is higher in African American (AA) and Hispanic populations relative to non-Hispanic whites (nHW), with larger differences for women compared to men of the same race. Given the public health importance of this issue, we sought to determine if AA and Hispanic women exhibit worse ADRD pathology compared to men of the same race and nHW women. We hypothesized that such differences may explain the discrepancy in ADRD prevalence.

**Methods:**

We evaluated 932 articles that measured at least one of the following biomarkers of ADRD pathology *in vivo* and/or post-mortem: beta-amyloid (Aß), tau, neurodegeneration, and cerebral small vessel disease (cSVD). Criteria for inclusion were: (1) mean age of participants >65 years; (2) inclusion of nHW participants and either AA or Hispanics or both; (3) direct comparison of ADRD pathology between racial groups.

**Results:**

We included 26 articles (Aß = 9, tau = 6, neurodegeneration = 16, cSVD = 18), with seven including sex-by-race comparisons. Studies differed by sampling source (e.g., clinic or population), multivariable analytical approach (e.g., adjusted for risk factors for AD), and cognitive status of participants. Aß burden did not differ by race or sex. Tau differed by race (AA < nHW), and by sex (women > men). Both severity of neurodegeneration and cSVD differed by race (AA > nHW; Hispanics < nHW) and sex (women < men). Among the studies that tested sex-by-race interactions, results were not significant.

**Conclusion:**

Few studies have examined the burden of ADRD pathology by both race and sex. The higher prevalence of ADRD in women compared to men of the same race may be due to both higher tau load and more vulnerability to cognitive decline in the presence of similar Aß and cSVD burden. AA women may also exhibit more neurodegeneration and cSVD relative to nHW populations. Studies suggest that between-group differences in ADRD pathology are complex, but they are too sparse to completely explain why minority women have the highest ADRD prevalence. Future work should recruit diverse cohorts, compare ADRD biomarkers by both race and sex, and collect relevant risk factor and cognitive data.

## Introduction

Alzheimer’s disease and Related Dementias (ADRD) are progressive neurodegenerative illnesses clinically characterized by difficulties with memory and other cognitive abilities such as language and executive function (Matthews et al., 2019). Presently, 47 million people worldwide suffer from ADRD (Babulal et al., 2019). As the proportion of individuals older than 65 grows, this figure is estimated to increase by roughly 10 million new cases each year (Babulal et al., 2019). In addition to growing in number, the population of those older than 65 is expanding to include more racial minorities. In the United States, African American (AA) and Hispanic populations are projected to see steep increases in the number of people diagnosed with ADRD over the next 40 years (Matthews et al., 2019). Currently, compared to non-Hispanic white (nHW) populations, AA are 2 times more likely and Hispanics 1.5 times more likely to be clinically diagnosed with ADRD (Alzheimer’s Association, 2019). When stratified by sex, women of these racial groups are at even higher risk of being diagnosed with ADRD compared to men (Matthews et al., 2019). In fact, there is emerging evidence that relative to all men and nHW women aged 65 and older, AA and Hispanic women have the first and second highest prevalence of ADRD, respectively (Matthews et al., 2019).

There are several race-associated factors that may explain these differences. AA and Hispanic populations are more likely than nHW to develop cardiometabolic diseases such as hypertension, diabetes, and obesity (Alzheimer’s Association, 2019). Such risk factors are linked to higher risk of AD largely through their effects on cerebral vascular integrity. Exposure to vascular risk factors increases the likelihood of cerebral small vessel disease, which is associated with both onset and progression of ADRD (Babulal et al., 2019). Additionally, minorities are less likely to have access to high quality food or exercise-friendly neighborhoods (Zlokovic et al., 2020); both good nutrition and exercise are vital for maintaining brain health throughout the lifespan and may help curb effects of AD pathology on cognition (Zlokovic et al., 2020).

Because prevalence of AD is greater among all women compared to men independent of race (Matthews et al., 2019), any race-related factor may additionally interact with those that are specific to sex. One proposed biological mechanism of greater ADRD risk among women is menopausal-related changes in endogenous estrogen production. Estrogen is thought to be neuroprotective against pernicious effects of ADRD pathology, so its rapid decrease during menopause may precipitate the development of AD (Fisher et al., 2018). Others have hypothesized that the ε4 allele of the APOE gene interacts with sex to confer greater risk of AD among women relative to men (Nebel et al., 2018), but the mechanisms of such effect modification are poorly understood. In addition to biological risk factors, it should also be noted that higher prevalence rates of ADRD in women relative to men may be due in part to survivor bias. That is, women have longer life expectancies than men of their same race (Arias, 2021) and age is the greatest risk factor for AD (Jack et al., 2015). Thus, compared to men, women may spend more time at high risk of being diagnosed with ADRD coupled with longer time spent with the disease (Andersen et al., 1999).

Many risk factors for ADRD overlap in women and minorities. Compared to men and nHW populations, both women and minorities, respectively, are at greater risk of psychosocial risk factors such as depressive symptoms (Barnes and Bennett, 2014; Nebel et al., 2018). Importantly, those who experience depressive symptoms in midlife are more likely to develop AD, potentially due to shared neural substrates and pathways for memory and stress hormone dysregulation, respectively (Nebel et al., 2018). Further, both post-menopausal women and minorities are affected by poorer sleep quality, which emerging evidence suggests is related to ADRD pathology clearance (Grandner et al., 2016; Nebel et al., 2018; Irwin and Vitiello, 2019).

Given these race- and sex-related discrepancies for risk factors of ADRD, it follows that we would expect to also find race- and sex- related discrepancies in ADRD pathology.

The hallmark pathological features of AD are extracellular plaques made of beta-amyloid (Aß) and intracellular neurofibrillary tangles comprised of hyperphosphorylated tau (Jack et al., 2013). While the biological cascade of events is complex, it is generally accepted that these proteins begin to aggregate decades prior to symptomatology and that Aß precedes tau which precedes neurodegeneration (Jagust, 2018). In the beginning stages of AD, Aß and tau aggregate in the parietal cortex and medial temporal lobe, respectively (Jagust, 2018). Progressively, these abnormal proteins begin to deposit in other areas of the brain, ultimately yielding greater atrophy and resultant worsening clinical symptoms.

The advent of *in vivo* biomarkers has been integral in uncovering the underlying mechanisms of AD. Brain Aß load can be estimated through positive correlation with amyloid positron emission tomography (PET) radiotracer uptake or negative correlation with cerebral spinal fluid (CSF) Aß-42 markers. Tau burden in the brain is positively correlated with both PET radiotracer retention and CSF-derived total- and phosphorylated-tau. In addition to these, cerebral Aß and tau pathology can be measured through blood-based markers (e.g., Aß42/Aß40 ratio, total-tau, and phosphorylated-tau), but efforts to improve the sensitivity of these biomarkers are ongoing (Zetterberg, 2019). Neurodegeneration is commonly estimated through magnetic resonance imaging (MRI). However, it can also be measured by CSF- or blood-derived neurofibrillary light (NfL) chain, which is correlated positively with axonal injury (Petzold, 2005; Olsson et al., 2016; Zetterberg, 2019). Of note, neuroimaging biomarkers like PET and MRI provide information regarding the severity of AD pathology as well as its topographical distribution. In contrast, CSF- and blood-based biomarkers reflect the severity of pathology, but do not provide insight into its topography.

Racially diverse and representative studies that examine ADRD pathology differences between racial groups are fairly limited and as a result, consensus surrounding race-related differences in Aß, tau, and neurodegeneration have not yet been reached (Babulal et al., 2019). In contrast, work examining sex-related differences in primarily nHW cohorts is more common. It has been reported that compared to men, women show similar levels of Aß (Ferretti et al., 2018), elevated tau (Buckley et al., 2019; Ossenkoppele et al., 2020), and less neurodegeneration (Jack et al., 2015; Sundermann et al., 2016; Ossenkoppele et al., 2020).

The overarching goal of this review was to examine sex-by-race differences in biomarkers of ADRD pathology. To do this, we conducted an Ovid MEDLINE search for studies that compare relevant AD biomarkers by race and sex-by-race. More specifically, we summarized race- and sex-by-race-related comparisons of Aß, tau, and neurodegeneration; this classification system is congruent with the National Institute on Aging and the Alzheimer’s Association’s proposed AT(N) framework (Jack et al., 2018), which categorizes research participants as neuropathologically normal or abnormal for A (Aß), T (tau), and N (neurodegeneration). In addition to these measures, we also included work related to cerebral small vessel disease (cSVD) as recent evidence suggests that vascular damage may affect other biological changes related to AD, including Aß clearance (Shaaban et al., 2017); further, cSVD is related to both race and sex, and thus may be important to understanding differing ADRD risk profiles among minority women (Jorgensen et al., 2018). In summary, based on existing literature, it appears that compared to nHW, minorities exhibit worse cSVD; compared to men, women present more tau. Thus, we hypothesized that a combination of more severe risk profiles for both cSVD and tau could explain why minority women have the highest prevalence of ADRD compared to the rest of the older adult population. Alternatively, it may be that pathology is similar in AA and Hispanic women compared to men of their same race and nHW populations, but that vulnerability to cognitive decline differs between groups.

## Materials and Methods

We used Ovid MEDLINE to retrieve articles for the narrative literature review through October 2020; line-by-line search terms are outlined in Supplementary Table 1.

Once we completed the search and removed duplicates, we screened articles for eligibility using titles and abstracts. During screening, we excluded articles for the following reasons: (1) the study was conducted in animals; (2) the study was conducted in a sample with a mean age less than 65; (3) the study did not include African Americans or Hispanics in the sample; (4) the study only conducted analyses adjusting for race; (5) the study sample included only one race; (6) the study did not include relevant biomarkers for AD; or (7) the study sample was comprised of individuals with other psychiatric or major illnesses or injuries.

Subsequent to title and abstract screening, we assessed full-text articles. We excluded articles if the study did not compare an AD biomarker by race (*n* = 55), if only one race was included in the sample (*n* = 1), or if the mean age of the sample was younger than 65 (*n* = 2).

**FIGURE 1:**
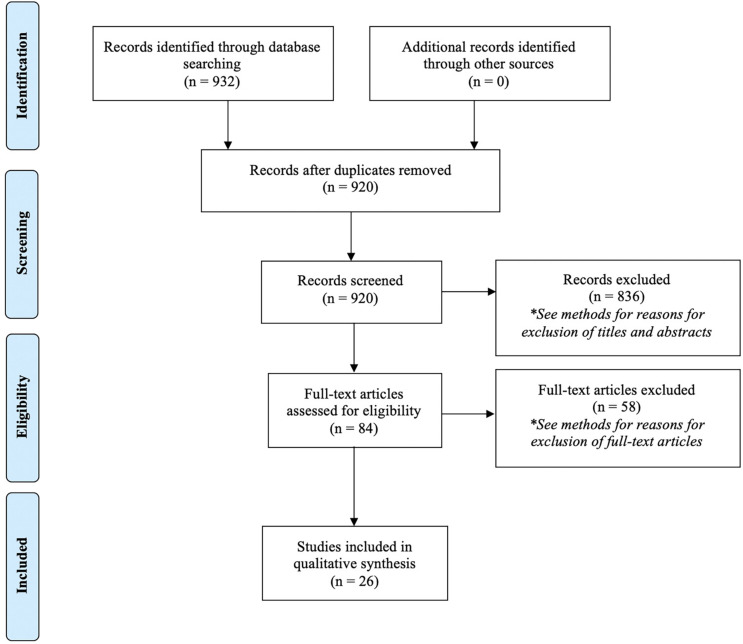
PRISMA flow diagram delineating the search for literature completed through October 2020.

A flow diagram outlining the search process is outlined in Figure 1, following PRISMA guidelines (Moher et al., 2009).

For each full-text article that we assessed, we noted the characteristics that are most likely to influence results: sampling source, multivariable analytical approach, and cognitive status of the participants.

Differing sampling sources may yield inconsistent results between two studies, particularly if one set of participants is recruited from an Alzheimer’s Disease Research Center (ADRC) and the other from the community. Compared to the rest of the population, ADRC-recruited participants are often younger, more educated, and more likely to have at least one APOE-ε4 allele (Farrer et al., 1997; Snitz et al., 2018). In other words, participants recruited from an ADRC are not necessarily representative of the population; it follows that results identified in such recruitment samples may not be identical to those from a population-based sample.

Additionally, the multivariable analytical approach can affect results if investigators do not account for factors that influence ADRD pathology. For example, older age is a risk factor for neurodegeneration (Jack et al., 2015). Thus, an imbalance in chronological age between comparison groups may lead to spurious between-group differences of neurodegeneration. Further, other health conditions or diseases that are outside of the nervous system, but can affect the brain, like diabetes and hypertension (Alzheimer’s Association, 2019), also necessitate consideration for statistical adjustment; more frequent occurrence in one of the comparison groups can again yield an apparent group difference in ADRD pathology.

Cognitive status of the participant is a proxy for ADRD pathology and as such, may lead to varying results between cross-sectional studies if the samples differ in this regard. That is, a sample of participants who are cognitively normal would likely only capture those with a low burden of ADRD pathology while that comprised of clinically-diagnosed AD patients would exhibit advanced stages of pathology. As such, these two cohorts, which represent different parts of the natural history of AD, are not comparable, and will likely not yield identical results.

## Results

**FIGURE 2:**
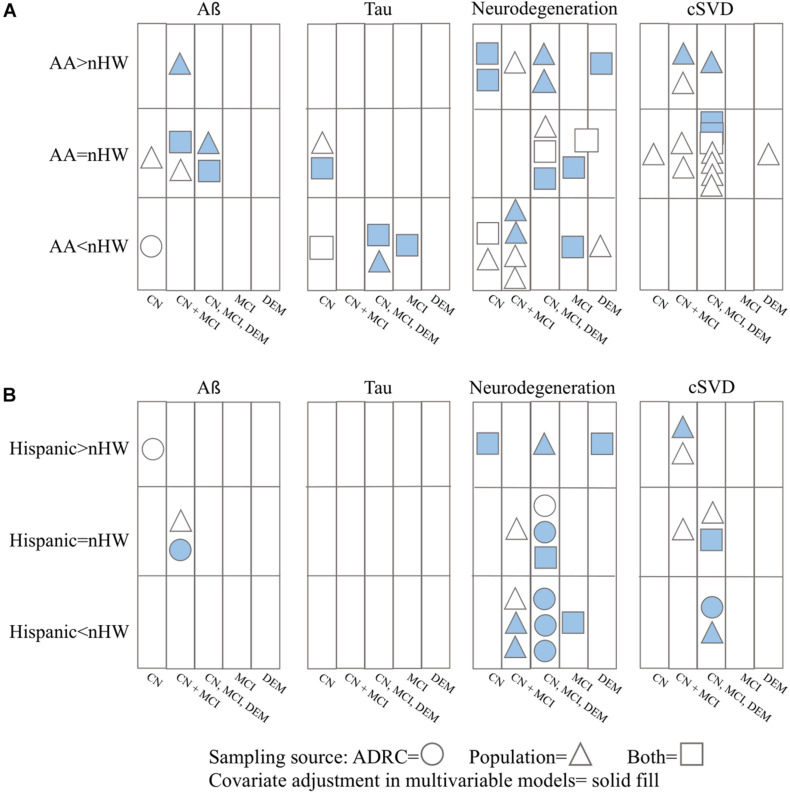
Summary of results for ADRD pathology differences between nHW and **(A)** African Americans and **(B)** Hispanics. CN = cognitively normal; MCI = mild cognitive impairment; DEM = dementia. Studies that did not report cognitive status or recruitment source of their participants are not included (*n* = 6).

In total, 26 articles were included for review. Of these, nine measured Aß, 6 measured tau, 16 measured neurodegeneration, and 18 measured cSVD. Seven articles additionally assessed sex-by-race differences. In all studies, biomarker comparisons were made cross-sectionally. A summary of the main findings surrounding race differences in ADRD biomarkers is shown in Figure 2; race- and sex-by-race-specific findings are outlined in Supplementary Table 2.

### Amyloid Biomarkers

Among nine studies examining amyloid biomarkers, three used CSF Aß-42 measurements (Howell et al., 2017; Garrett et al., 2019; Morris et al., 2019), six used PET imaging (Gu et al., 2015; Gottesman et al., 2016; Duara et al., 2019; Morris et al., 2019; Amariglio et al., 2020; Han et al., 2020), and one examined post-mortem tissue (Riudavets et al., 2006). Sources of recruitment included clinical and community settings; notable population-based samples included the Atherosclerosis Risk in Communities (ARIC) study, the Harvard Aging Brain Study (HABS), and the Washington Heights Inwood Columbia Aging Project (WHICAP). Participant cognitive status spanned normal cognition, mild cognitive impairment (MCI), and AD. Sample sizes ranged from 135 to 1255. Average ages ranged from 65 to 84 years. Two of these studies examined sex-by-race related differences, the findings of which are described in the section “Sex Differences in Amyloid Biomarkers by Race.” In the following section, we outline race-related differences for both male and female participants combined.

#### Racial Differences in Amyloid Biomarkers

All (Riudavets et al., 2006; Gu et al., 2015; Howell et al., 2017; Duara et al., 2019; Garrett et al., 2019; Morris et al., 2019; Amariglio et al., 2020) but two (Gottesman et al., 2016; Han et al., 2020) studies reported no significant racial differences in Aß burden.

Of those that measured CSF Aß-42 concentrations, one community-based study of 1255 adults ranging from normal cognition, MCI, and AD reported no significant differences between AA and nHW after adjusting for age, sex, APOE-ε4 allele status, education, clinical status, body mass index (BMI), family history of AD, and CSF drift variables (Morris et al., 2019). Two other studies (Howell et al., 2017; Garrett et al., 2019), both of which recruited from ADRC and community, similarly reported no significant CSF Aß-42 differences between AA and nHW. One of these studies included participants with cognitive status ranging from normal cognition to AD (Howell et al., 2017) and the other including only those with normal cognition and MCI (Garrett et al., 2019); results from both studies remained non-significant after stratifying by cognitive status and/or adjusting for covariates.

Brain PET studies also largely reported no significant racial differences in Aß radiotracer retention for either AA or Hispanic populations relative to nHW. Morris et al. (2019) reported global ^[11–C]^Pittsburgh Compound-B (PiB) standardized uptake value ratios (SUVR) that were not statistically different in AA compared to nHW in participants ranging in cognitive status (normal cognition, MCI, AD) and recruited from the community; this comparison was made after adjustment for age, sex, APOE-ε4 allele status, education, clinical status, BMI, and family history of AD. Similarly, work from the community-based HABS revealed that among 296 cognitively normal participants, AA did not statistically differ in average cortical PiB SUVR from nHW (Amariglio et al., 2020). One study that included 116 dementia-free AA, Hispanic, and nHW participants from WHICAP reported similarly null racial differences such that there were no significant differences in the proportion of participants categorized as visually ^[18–F]^Florbetaben (FBB) SUVR positive by race (Gu et al., 2015); 32% of AA, 31% of Hispanics, and 40% of nHW were FBB positive. In a study that recruited 159 Hispanic and nHW participants ranging in cognitive status (cognitively normal, MCI, dementia) from an ADRC, no significant racial differences were detected in FBB SUVR (Duara et al., 2019). Race additionally did not significantly predict FBB SUVR in multivariable linear regression models adjusted for age, Mini Mental State Examination (MMSE) score, and APOE-ε4 allele status.

One post-mortem study that did not report clinical status of participants detected no racial differences in Aß burden among 100 participants. Using this community-based sample that included AA and nHW brains, Riudavets et al. (2006) reported that in each racial sample, about two-thirds of participants exhibited Aß plaques. Further, logistic regression analyses revealed that race did not significantly predict Aß plaque or Aß angiopathy presence.

In contrast to these findings, the ARIC study found that among 329 participants without dementia, AA had 2-fold greater odds of global ^[18–F]^Florbetapir (FBP) SUVR positivity relative to those who were nHW (Gottesman et al., 2016). This relationship was not explained by age, sex, education, APOE-ε4 allele status, hypertension, diabetes, cognitive status, or white matter hyperintensity (WMH) volume. Similarly, an ADRC-based study of 85 participants with normal cognition reported that compared to nHW, a higher percentage of Hispanics, but not AA, met the threshold for FBP positivity (Han et al., 2020). Within the study sample, 50% of Hispanics, 30% of AA, and 44% of nHW participants were classified as FBP positive. However, no formal statistical test assessed if these group differences were significant.

Longitudinal analyses from HABS revealed that in those with elevated PiB SUVR at baseline, AA participants showed annual composite cognitive decline that was 0.05 standard deviations faster compared to nHWs (Amariglio et al., 2020). Although small in absolute terms, this difference was statistically significant even after adjustment for age, sex, and baseline Preclinical Alzheimer’s Cognitive Composite score. Additionally, investigators from WHICAP found that higher FBB SUVR was associated with small but significant yearly decline in average cognition, language, and executive function in AA, but not nHW participants (Gu et al., 2015).

#### Sex Differences in Amyloid Biomarkers by Race

Within these nine studies of Aß comparisons between racial groups, two also measured sex-by-race differences; women generally exhibited higher or similar burden of amyloid independent of race. The ARIC study found that in both AA and nHW participants, women were 1.7 times more likely than men to be globally FBP positive after adjustment for age, race, education, APOE-ε4 allele status, hypertension, and diabetes (Gottesman et al., 2016). Additionally, when stratified by race and sex, Riudavets et al. (2006) found that a greater proportion of AA and nHW women displayed Aß plaques relative to men of their respective race. However, sex was not a significant predictor of Aß plaques in linear models adjusted for age. Results for amyloid angiopathy were less straightforward. Among nHW participants, a greater proportion of women were dichotomously classified as exhibiting amyloid-angiopathy. However, among AA participants, the number of participants showing such pathology was roughly equal.

#### Summary and Synthesis

Overall, these studies do not provide strong evidence for racial differences in Aß deposition. While the ARIC study did report that AA had significantly greater Aß burden than nHW, this may have been the result of geographic exposures. Most of the AA participants in ARIC were from the Jackson, Mississippi site (Gottesman et al., 2016); FBP SUVRs in AA participants at the other two sites were 6% higher than all nHW participants, but 4% lower than those at the Jackson site. Thus, the true racial difference may be overestimated in this sample. These results may additionally be biased by the effects of smoothing the PET images to a common resolution. That is, amyloid was quantified using FBP, an Aß radiotracer with comparatively narrower dynamic range than others on account of its higher non-specific white matter retention (Wong et al., 2010; Landau et al., 2013). Others have reported that smoothing exacerbates the effect of white matter retention and further compresses the range of FBP (Landau et al., 2013). Assuming that AA populations exhibit worse neurodegeneration (and thus, less white matter) than nHW (see section “Neurodegeneration Biomarkers”), it follows that compared to nHW, AA participants’ PET images may exhibit relatively less FBP white matter binding, positively skewing SUVRs. Similar to the ARIC study, Han et al. (2020) found greater prevalence of FBP positivity in Hispanic participants compared to nHW. However, because this difference was not formally tested for statistical significance, it should be interpreted cautiously.

Results from these studies similarly did not yield strong evidence for sex differences in Aß in either direction. The findings are in contrast to many recent studies that have largely found no differences between men and women in terms of Aß burden (Mielke et al., 2012; Altmann et al., 2014; Buckley et al., 2019). However, it should be noted that most studies that have measured sex differences in Aß have done so in largely white samples. As such, though few in number, these findings from racially diverse epidemiological cohorts indicate that sex differences in Aß among other races may differ from those identified in nHW participants.

Two articles included in this review found that compared to nHW, AA exhibit worse cognition in the presence of comparable Aß load (Gu et al., 2015; Amariglio et al., 2020). Before further discussion of these studies, it should be noted that results may be influenced by pathologies that, compared to Aß, are more strongly associated with cognitive impairment, but were not accounted for in analyses. Such pathologies include tau and Lewy bodies (Schneider et al., 2012; Hedden et al., 2013; LaPoint et al., 2017; Jansen et al., 2018; Maass et al., 2018), the latter of which may be more common in AA with AD (Barnes et al., 2015). Despite this limitation, evidence still suggests that in response to Aß, AA populations potentially possess relatively less cognitive resilience or worse cognitive adaptability to neuropathological insult (Arenaza-Urquijo and Vemuri, 2018). These studies did not report race-related differences in cognition separately for men and women, but instead combined data across sexes. However, previous work has found that compared to men, women exhibit worse cognitive resilience in the presence of similar burden of AD pathology, including Aß (Gamberger et al., 2017; Koran et al., 2017). Taken together with results from studies on racial differences, it is possible that minority women face a double burden for risk of accelerated cognitive decline in the presence of Aß.

### Tau Biomarkers

A total of six studies measured tau, three of which did so with CSF (Howell et al., 2017; Garrett et al., 2019; Morris et al., 2019), one with blood (Rajan et al., 2020), one with PET imaging (Lee et al., 2018), and one with post-mortem tissue (Riudavets et al., 2006). Study samples were recruited from ADRC and population-based settings, the latter of which notably included The Chicago Health and Aging Project (CHAP) and HABS. Participant clinical status included normal cognition, MCI, and AD. Sample sizes ranged from 146 to 1327. The average age of cohorts ranged from 65 to 76 years. No studies included Hispanic participants. Sex-by-race differences were reported in only one of these studies, which is summarized in the section “Sex Differences in Tau Biomarkers by Race.”

#### Racial Differences in Tau Biomarkers

Overall findings for racial differences in tau burden were mixed. Among the CSF-derived tau studies, one ADRC- and community-based sample of 362 participants reported racial differences in MCI, but not cognitively normal, participants (Garrett et al., 2019). Among MCI participants, AA displayed less total- and phosphorylated-tau on average relative to nHW independent of age, sex, education, family history of AD, BMI, Montreal Cognitive Assessment (MoCA), hypertension, diabetes, and income. However, in cognitively normal participants, AA and nHW did not differ in either total- or phosphorylated-tau after covariate adjustment. In another ADRC- and community-based study comprised of 135 participants with either normal cognition, MCI, or dementia, investigators found racial differences for both total- and phosphorylated-tau such that AA exhibited less burden than nHW (Howell et al., 2017); this difference persisted after adjustment for cognitive function, age, sex, APOE-ε4 allele status, ABCA7 risk allele status, Aß-42, hypertension, diabetes, and WMH volume. A third study found racial differences in tau independent of cognitive status (Morris et al., 2019); in a cohort of 1255 cognitively normal, MCI, and AD participants, AA exhibited average total- and phosphorylated-tau concentrations that were less than nHW. This difference was not explained by age, sex, APOE-ε4 status, education, clinical status, family history of AD, BMI, or CSF drift variables.

In contrast to the above findings, two other studies reported no racial differences in tau load for either blood-based (Rajan et al., 2020) or PET-based (Lee et al., 2018) markers. CHAP, which included blood-based measurements in 1,327 participants with normal cognition, found no significant difference in total-tau between AA and nHW (Rajan et al., 2020). In a HABS subset of 146 participants whose cognitive status were not reported, investigators similarly did not find significant differences between AA and nHW participants in ^[18–F]^Flortaucipir (FTP) SUVR in the amygdala, entorhinal, fusiform, or inferior temporal regions-of-interest after adjustment for age, sex, education, and Aß burden (Lee et al., 2018). Of exception, AA participants showed greater FTP SUVR in the choroid plexus and hippocampus, but the authors note that this was likely due to off-target binding of the radiotracer and spill-in, respectively.

In a post-mortem study that did not report the overall difference between AA and nHW in tau lesions, AA race was not significantly associated with odds of tau lesions after adjusting for age in a sample of 100 participants (Riudavets et al., 2006). This study also did not report the cognitive status of participants.

#### Sex Differences in Tau Biomarkers by Race

Of the studies that measured tau differences between racial groups, one also measured and reported sex differences. In a post-mortem study that did not report the cognitive status of participants, Riudavets et al. (2006) reported greater severity of tau lesions in nHW women compared to men such that 96% of nHW women exhibited tau lesions whereas only 88% of men did so. In contrast, 96% of both AA women and men displayed tau lesions. When broken down by region, results differed slightly such that independent of race, a greater percentage of women showed more advanced tauopathy. Importantly, none of the comparisons made in this study were done so with formal statistical analysis. Thus, they should be interpreted cautiously.

#### Summary and Synthesis

Studies found that tau burden among AA populations was either less than or similar to that of nHW. These mixed findings between studies may be due in part to the age of participants. *In vivo* studies that reported less relative tau burden in AA included participants who were younger (average age range: 65–70 years) (Howell et al., 2017; Garrett et al., 2019; Morris et al., 2019) compared to those that reported no differences (average age range: 73–76 years) (Lee et al., 2018; Rajan et al., 2020). Thus, it may be that the analyses that found no racial differences included participants with more advanced tauopathy either due to age (Lowe et al., 2018) or AD progression. Such discrepancies between studies warrant that future work examine whether or not racial differences in tau pathology at the beginning of late adulthood and/or AD persist through the natural history of the disease. Taken together with the lack of such differences in the post-mortem study included in this review (Riudavets et al., 2006), it is possible that while younger AA populations exhibit lesser tau accumulation in relation to nHW, at some point during the course of AD, or as individuals age, the burden of tau converges to be comparable among the two racial groups. In other words, compared to nHW, in AA tau accumulation may begin later and accrue more rapidly. Findings from Howell et al. (2017) support this hypothesis; this group reported that racial differences were greatest among cognitively normal participants, but in those with MCI and AD, the gap in tau burden progressively decreased.

The sex differences by race in post-mortem tau burden in Riudavets et al. (2006) may also be influenced by age and/or disease progression. At the time of autopsy, on average, women were older compared to men of their respective races. Thus, it is reasonable that a greater proportion of women would show evidence of a more advanced disease stage. Women were much older than men particularly among the nHW participants, which may also have influenced the more pronounced sex-by-race differences in pathology among nHW participants, comparatively. These limitations notwithstanding, these sex differences are consistent with previous work conducted in nHW populations (Buckley et al., 2019; Ossenkoppele et al., 2020). Future work should apply either age stratification methods or more consistent age-adjusted analyses to further investigate sex differences in tau among AA populations.

### Neurodegeneration Biomarkers

Among the 16 studies that examined neurodegeneration, 2 measured NfL (Howell et al., 2017; Rajan et al., 2020), 4 measured total brain volume (Minagar et al., 2000; Brickman et al., 2008; DeCarli et al., 2008; Aggarwal et al., 2010), 8 measured hippocampal and entorhinal volumes (DeCarli et al., 2008; Zahodne et al., 2015; Howell et al., 2017; Burke et al., 2018; Duara et al., 2019; Garrett et al., 2019; Morris et al., 2019; Arruda et al., 2020), 4 measured cortical thickness (Zahodne et al., 2015; McDonough, 2017; Rizvi et al., 2018; Arruda et al., 2020), and 4 measured lateral ventricle size (Minagar et al., 2000; Shadlen et al., 2006; Brickman et al., 2008; Burke et al., 2018). Participants in these studies ranged from cognitively normal, MCI, and AD and were recruited from both ADRC and community settings. Of those recruited from the community, notable study samples included The Cardiovascular Health Study (CHS), CHAP, HABS, and WHICAP. Study sample sizes ranged from 135 to 2786 and average ages ranged from 65 to 80 years. Among these, 2 examined sex-by-race-associated differences, which are described in the section “Sex Differences in Neurodegeneration Biomarkers by Race”; sections “Racial Differences in NfL Concentrations” through “Racial Differences in Lateral Ventricle Size” report race-related differences for both male and female participants combined.

#### Racial Differences in NfL Concentrations

In an ADRC- and community-based sample comprised of 135 participants, investigators reported that CSF-derived NfL levels were lower in cognitively normal AA compared to nHW after adjustment for age, sex, APOE-ε4 allele status, ABCA7 risk allele status, Aß-42, hypertension, diabetes, and WMH volume (Howell et al., 2017). However, among those with cognitive impairment, NfL levels did not differ between races after covariate adjustment. Analyses from CHAP revealed in a sample 1,327 participants with normal cognition that blood plasma-derived concentrations of NfL were lower in AA relative to nHW across the entire cohort (Rajan et al., 2020). However, this between-group difference was not formally tested for statistical significance.

#### Racial Differences in Total Brain Volumes

A pattern emerged among studies that measured total brain volume such that investigators consistently reported no racial differences. Analyses from CHAP reported no significant difference in total brain volumes between AA and nHW participants in a sample of 575 participants who ranged from normal cognition to dementia (Aggarwal et al., 2010). DeCarli et al. (2008) similarly found that AA and Hispanic participants did not display significantly different average brain volumes compared to nHW in a sample of 401 participants with normal cognition, MCI, and AD; this was after adjustment for age, sex, education, and cognitive status. Finally, in a study that included 144 participants diagnosed with AD, Minagar et al. (2000) found no difference in visually-assessed cortical neurodegeneration between Hispanics and nHW after adjusting for age, gender, education, MMSE score, and disease duration.

Of exception, in a subset of WHICAP with 702 cognitively normal and MCI participants, Brickman et al. (2008) reported that AA and Hispanic participants both exhibited mean total brain volumes that were larger than that of nHW after adjusting for age, sex, and vascular disease history.

#### Racial Differences in Hippocampal and Entorhinal Volumes

Racial differences in hippocampal and entorhinal volumes among AA populations were mixed across six studies. One WHICAP subset that included 638 participants ranging from normal cognition to MCI found that AA displayed greater average hippocampal volume compared to nHW, with a small effect size (Cohen’s *d*) of 0.23 (Zahodne et al., 2015). Additionally, DeCarli et al. (2008) reported that in an ADRC- and community-based cohort of 401 participants, among those with MCI, AA had larger hippocampal volumes after adjustment for age, sex, and education.

Three other studies reported smaller regional volumes in AA relative to nHW populations. Morris et al. (2019) reported that in a cohort of 1255 community-recruited participants with varying cognitive status (normal, MCI, and AD), AA displayed smaller average hippocampal volumes than that of nHW after covariate adjustment. However, this finding was influenced by family history of dementia. When stratified, AA participants with family history of dementia had smaller hippocampal gray matter volumes (GMV) than nHW with family history of dementia. In those without such family history, no racial differences in hippocampal GMV were detected. Similarly, DeCarli et al. (2008) reported that among those with normal cognition and dementia, AA had smaller hippocampal volumes; this could not be explained by adjustment for age, sex, and education. In another cohort of 362 participants recruited from both ADRC and community, cognitively normal AA participants’ average hippocampal volumes were smaller than that of nHW independent of age, sex, educational level, BMI, family history of AD, MoCA score, hypertension, diabetes, and income (Garrett et al., 2019).

In contrast to those described above, two other studies found no significant differences in hippocampal volumes between AA and nHW participants. In one study of 135 participants ranging from cognitively normal to AD, Howell et al. (2017) found that race had no effect on hippocampal volume. Garrett et al. (2019) also reported that average hippocampal volumes in AA with MCI were not statistically different than nHW after adjusting for covariates.

Studies that included Hispanic populations also reported mixed findings. Four articles reported greater medial temporal regions in Hispanics compared to nHW. In a subset from WHICAP that only included participants with normal cognition and MCI, Hispanic participants displayed greater average hippocampal volumes relative to nHW with an effect size (Cohen’s *d*) of 0.28 (Zahodne et al., 2015). Among those with MCI in a study of 401 participants that recruited from both the ADRC and community, Hispanic participants exhibited greater hippocampal volumes relative to nHW after adjusting for age, sex, and education (DeCarli et al., 2008). One ADRC-based study with 226 participants ranging from cognitively normal to AD found that compared to nHW, Hispanic participants exhibited greater average hippocampal volumes (Cohen’s *d* = 0.30) and entorhinal volumes (Arruda et al., 2020). This group additionally reported that Hispanic race was associated with 0.12 mm^3^ greater hippocampal volume and 0.13 mm^3^ greater entorhinal volume independent of age, gender, functional activities questionnaire score, and MoCA score. In another ADRC-based study of 165 participants with normal cognition, MCI, and dementia, Hispanics showed greater hippocampal and entorhinal volumes relative to nHW after adjusting for age, depression, and Geriatric Depression Scale score (Burke et al., 2018).

Two articles reported patterns that were in contrast to the above findings. One ADRC-based study that included 159 participants with normal cognition, MCI, and AD found no significant difference in hippocampal volumes between Hispanic and nHW participants (Duara et al., 2019). Similarly, DeCarli et al. (2008) reported that among those with normal cognition or dementia, Hispanic participants displayed smaller hippocampal volumes relative to nHW after adjustment for age, sex, and education.

#### Racial Differences in Cortical Thickness

Findings generally suggested that compared to nHW populations, AA display smaller cortical thickness. In WHICAP, across 519 participants with normal cognition, MCI, or dementia, investigators found that after adjusting for age, education, and intracranial volume, AA exhibited smaller global cortical thickness compared to nHW participants (Rizvi et al., 2018). In another WHICAP subset that included 638 participants with normal cognition and MCI, AA displayed smaller AD-composite cortical thickness relative to nHW participants (Zahodne et al., 2015); this difference yielded an effect size (Cohen’s *d*) of 0.23. An investigation of 284 HABS participants revealed a similar pattern such that after participants were propensity score-matched using age, sex, education, verbal IQ, Aß burden, and white matter hypointensity presence, bootstrap ratios found 12 of 20 AD-signature cortical thickness regions that were significantly smaller in AA participants (McDonough, 2017).

Results regarding cortical thickness in Hispanic populations relative to nHW were less consistent. Rizvi et al. (2018) reported that in a WHICAP cohort of participants ranging from normal cognition to dementia, relative to nHW, Hispanic participants displayed smaller average global cortical thickness after adjustment for age, education, and intracranial volume. However, in another WHICAP analysis that only included participants with normal cognition or MCI, AD-composite cortical thickness was almost identical in nHW and Hispanic participants (Zahodne et al., 2015). Similarly, in an ADRC-based study that included 226 cognitively normal, MCI, and AD participants, Hispanic participants did not statistically differ from nHW in average entorhinal cortex thickness (Arruda et al., 2020). This study additionally reported that in a linear regression model adjusted for age, sex, education, functional activity questionnaire score, and MoCA score, Hispanic ethnicity was not associated with entorhinal cortex thickness.

#### Racial Differences in Lateral Ventricle Size

Studies typically reported that compared to nHW, minorities displayed significantly smaller lateral ventricles. In a subset of 2786 participants from CHS, investigators found that in those without dementia, 8% of the AA sample and 15% of the nHW sample exhibited large ventricular volumes (Shadlen et al., 2006). This pattern was similar among those with dementia as 16% of AA and 32% of nHW exhibited large ventricular volumes. In the WHICAP cohort across 702 participants with normal cognition and MCI, compared to being nHW, AA and Hispanic race was associated with smaller lateral ventricle volumes independent of age, sex, and vascular disease history (Brickman et al., 2008). Additionally, in an ADRC-based study of 165 participants, Hispanics exhibited left and right ventricle volumes that were smaller in cognitively normal, MCI, and dementia participants, compared to nHW participants with corresponding diagnoses (Burke et al., 2018)*;* this was after adjustment for age, education, and Geriatric Depression Scale score. Similarly, Minagar et al. (2000) reported in a study with 144 Hispanics and nHW diagnosed with AD that ventricular size was smaller in Hispanics relative to nHW after covariate adjustment.

#### Sex Differences in Neurodegeneration Biomarkers by Race

Of the studies that examined racial differences in neurodegeneration, two measured sex-by-race differences. These studies suggested a sex difference such that women generally displayed less neurodegeneration relative to their male counterparts of the same race. For example, in a subset of 702 cognitively normal and MCI participants in the WHICAP cohort, investigators found that when stratified by race, total brain volume was larger in women compared to men in nHW, AA, and Hispanics (Brickman et al., 2008). Despite this, a sex-by-ethnicity interaction for total brain volume was not significant. This group also reported that female sex was associated with smaller ventricles independent of race. Arruda et al. (2020) reported in an ADRC-based study among 226 Hispanic and nHW participants that female sex was associated with greater hippocampal volume independent of age, education, ethnicity, functional activities questionnaire scores, and MoCA scores. This relationship was found in both the entire cohort of cognitively normal, MCI, and AD participants as well as in a subsample limited to only those who were non-demented. However, this group also found that sex did not predict entorhinal cortex volume or thickness after covariate adjustment.

#### Summary and Synthesis

These studies generally reported similar amounts of global neurodegeneration in AA and Hispanic populations relative to nHW. This was consistent across studies that measured neurodegeneration using NfL concentrations (Howell et al., 2017) and total brain volumes (Minagar et al., 2000; DeCarli et al., 2008; Aggarwal et al., 2010). Still, two studies reported lesser NfL burden in AA participants relative to nHW (Howell et al., 2017; Rajan et al., 2020). However, because neither analysis adjusted for BMI, which is greater among AA populations compared to nHW (Hales et al., 2020) and negatively correlated with NfL concentration (Manouchehrinia et al., 2020), these estimates may be biased by such unmeasured confounding.

It should additionally be noted that one analysis from WHICAP found thinner global cortical thickness values in AA and Hispanic participants compared to nHW after controlling for age, education, and intracranial volume (Rizvi et al., 2018). Because this analysis was conducted in a large population-based study with higher MRI resolution and more precise segmentation methods relative to the studies that reported no racial differences in global neurodegeneration, it follows that this may be a more precise estimate of the true racial differences.

The discrepant findings between studies that measured neurodegeneration in regions characteristic of AD pathology in AA populations may be due to differences in study samples and methodology. For example, the articles that reported no differences in AD-specific regional volumes or cortical thicknesses recruited their samples at least in part from ADRCs (Howell et al., 2017; Garrett et al., 2019). Because participants recruited from ADRCs are generally healthier than the rest of the population, and thus, may exhibit better brain health, brain region size differences may have been skewed toward the null. Other investigators conducted their studies using either lower resolution MRIs (Brickman et al., 2008; DeCarli et al., 2008) or without adjusting for confounders in their analyses (Shadlen et al., 2006; Zahodne et al., 2015). However, studies that recruited participants from the community, used high resolution MRI with precise segmentation methods, and adjusted for covariates reported smaller AD-specific brain regions among AA participants compared to nHW (McDonough, 2017; Morris et al., 2019). In consideration with results surrounding global neurodegeneration, this pattern suggests that differences in the size of brain regions between AA and nHW populations may be characteristic of those affected by AD. However, longitudinal neuroimaging studies should examine if such racial differences exist due to preclinical brain region sizes, differences in brain resilience (how the brain structurally or functionally copes with neuropathological insult (Arenaza-Urquijo and Vemuri, 2018)), or both.

In contrast to results examining regional brain sizes in AA populations, studies generally suggested that the brain regions that are affected by AD are larger or similarly-sized in Hispanics compared to nHW. These findings were consistent among the studies that did not adjust for covariates (Minagar et al., 2000; Brickman et al., 2008; Zahodne et al., 2015; Duara et al., 2019) and those that did (DeCarli et al., 2008; Burke et al., 2018; Arruda et al., 2020). Of the studies that reported confounder-adjusted results, participants ranged in cognitive status. Based on these results, it is possible that Hispanics exhibit larger preclinical medial temporal lobe sizes compared to nHW, which contribute to racial differences in patterns of neurodegeneration across the natural history of AD. However, race-specific brain resilience mechanisms should additionally be examined.

Findings related to sex differences indicated that women exhibit less neurodegeneration compared to men independent of race. This is consistent with previous results conducted in largely white samples (Jack et al., 2015; Sundermann et al., 2016; Ossenkoppele et al., 2020). This sex-specific finding was most consistent among Hispanic women. Taken together with results surrounding racial differences in neurodegeneration, it is possible that compared to their aging counterparts, Hispanic women exhibit more brain resilience to AD pathology. This may partially explain the higher prevalence of AD among Hispanic women relative to nHW. Hispanic women have higher life expectancies than nHW women (Arias et al., 2015; Arias, 2016) and the greatest risk factor for AD is advanced age (Jack et al., 2015). As such, it may be that the brains of Hispanic women initially fare better in the presence of AD-related neuropathological insults relative to nHW women, but as a result, Hispanic women live longer to experience more time at high risk of developing AD.

### Cerebral Small Vessel Disease Biomarkers

Of the 18 studies that examined cerebral small vessel disease, 12 measured white matter hyperintensities (Minagar et al., 2000; Shadlen et al., 2006; Brickman et al., 2008; DeCarli et al., 2008; Aggarwal et al., 2010; Zahodne et al., 2015; Gottesman et al., 2016; Howell et al., 2017; Burke et al., 2018; Della-Morte et al., 2018; Rizvi et al., 2018; Amariglio et al., 2020) and eight measured ischemic lesions and other infarcts (Riudavets et al., 2006; DeCarli et al., 2008; Wright et al., 2008; Aggarwal et al., 2010; Wiegman et al., 2014; Zahodne et al., 2015; Morris et al., 2019). These studies recruited cognitively normal, MCI, and dementia participants from both ADRC and community. Notable population-based study samples included The Northern Manhattan Study (NOMAS), ARIC, CHAP, CHS, HABS, and WHICAP. Sample sizes ranged from 135 to 2786 and average ages of cohorts ranged from 70 to 80 years. Two of these studies examined sex-related differences, and are reported in the section “Sex Differences in cSVD Biomarkers by Race;” sections “Racial Differences in White Matter Hyperintensities” through “Racial Differences in Ischemic Lesions and Other Infarcts” report race-related differences for both male and female participants combined.

#### Racial Differences in White Matter Hyperintensities

These studies generally reported that relative to nHW, AA populations display significantly greater WMH burden whereas Hispanic populations display significantly less WMH burden.

Of the studies included, four reported significantly higher WMH volumes in AA relative to nHW populations, three of which were conducted in subsets of WHICAP. Among one such WHICAP subset of 519 participants who ranged from cognitively normal to AD, racial differences were detected between AA and nHW participants such that relative to nHW and Hispanic participants, AA showed greater average WMH volumes throughout the brain after controlling for age, education, and intracranial volume (Rizvi et al., 2018). AA additionally exhibited relatively greater average WMH volumes in the frontal, temporal, parietal, and occipital regions after covariate adjustment. In another WHICAP analysis that included 638 participants with either normal cognition or MCI, AA displayed mean WMH volume that was larger than that of nHW (Zahodne et al., 2015); the effect size (Cohen’s *d*) of this differences was 0.49. Finally, in the third WHICAP subset, among 702 participants with normal cognition and MCI, relative to nHW race, being AA was associated with greater WMH volumes independent of age, sex, and vascular disease (Brickman et al., 2008). In addition to these findings from WHICAP, another epidemiological study, NOMAS, reported that compared to nHW, mean WMH volume in AA participants was larger after adjustment for age, sex, health behaviors, BMI, and vascular risk factors (Della-Morte et al., 2018). This cohort was comprised of 1229 older adults whose cognitive status were not reported.

In contrast, six other studies reported no racial differences in WMH between AA and nHW populations. A study from ARIC found that in a subset of 329 dementia-free participants, AA exhibited average WMH volumes that were not statistically different compared to nHW (Gottesman et al., 2016). Additionally, DeCarli et al. (2008) found in an ADRC- and community-based sample of 401 cognitively normal, MCI, and dementia participants that AA or Hispanic race did not significantly predict WMH volume in a model that also included age, gender, education, and cognitive status. Similarly, in a subsample of 296 cognitively normal HABS participants, AA had average WMH volume that was not statistically different from nHW (Amariglio et al., 2020). Another ADRC- and community-based study of 135 cognitively normal, MCI, an dementia participants found no statistically significant differences between AA and nHW WMH volumes (Howell et al., 2017). Among 575 CHAP participants, AA and nHW participants did not significantly differ in WMH volume (Aggarwal et al., 2010). Finally, using data from 2786 CHS participants, one study found that among those with dementia, the same proportion of AA and nHW participants had high WMH grades (Shadlen et al., 2006), but this was not tested for statistical significance.

In studies that measured WMH in Hispanic populations, three reported lesser burden among Hispanics compared to nHW. One analysis using data from 519 WHICAP participants found that in those ranging from cognitively normal to AD, Hispanics exhibited smaller total WMH volume compared to nHW and AA populations (Rizvi et al., 2018); this finding was independent of age and education. This study also found that after covariate adjustment, Hispanics displayed relatively smaller average WMH volumes for the frontal, temporal, and occipital regions. Burke et al. (2018) found in an ADRC-based cohort of 165 participants ranging from cognitively normal to dementia that race was significantly associated with visually-rated WMH volume such that Hispanics exhibited smaller WMH volumes relative to nHW. This was not explained by age, education, or geriatric depression score. This group additionally reported that after covariate adjustment, cognitively normal Hispanic participants displayed smaller WMH volumes relative to nHW; the magnitude of this difference increased with worsening cognition. Additionally, among 1229 older adults whose cognitive status were not reported in NOMAS, investigators reported that compared to nHW, mean WMH volume in Hispanic participants was larger after adjustment for age, sex, health behaviors, BMI, and vascular risk factors (Della-Morte et al., 2018).

In contrast to these findings, two studies that included cognitively normal and MCI participants from WHICAP reported greater WMH volumes among Hispanics relative to nHW. One of these subsets, which included 638 participants, reported that Hispanics displayed mean WMH volume that larger than that of nHW, with a detected effect size (Cohen’s *d*) of 0.28 (Zahodne et al., 2015). The other subset reported greater WMH volume among Hispanics relative to nHW independent of age, sex, and vascular disease (Brickman et al., 2008).

Finally, one group reported no difference in visually-assessed WMH burden between Hispanics and nHW in a sample of 144 participants with AD reported (Minagar et al., 2000). Further, Hispanic race did not predict WMH burden after controlling for age, education, gender, MMSE score, and AD duration.

#### Racial Differences in Ischemic Lesions and Other Infarcts

Studies of comparisons by race for ischemic lesions and infarcts consistently reported a lack of statistically significant differences between nHW and either AA or Hispanic participants.

In studies that measured such biomarkers among AA and nHW populations, four found no racial differences. Among 575 CHAP participants who were classified as either having or not having dementia, no significant difference was detected between AA and nHW participants exhibiting more than one infarct (Aggarwal et al., 2010). Another community-based sample with 1255 participants ranging from cognitively normal to AD found that the proportion of AA participants who displayed lesions was not statistically different than that of nHW (Morris et al., 2019). DeCarli et al. (2008) found in an ADRC- and community-based cohort of 401 participants that subcortical infarcts did not vary significantly between AA and nHW after adjustment for age, gender, education, and cognitive status. Additionally, in a subset of 638 WHICAP participants that only included those with normal cognition and MCI, the proportion of AA participants with infarcts did not differ significantly than that of nHW (Zahodne et al., 2015). In another WHICAP subset of 243 participants ranging from cognitively normal to dementia, Wiegman et al. (2014) reported that the same proportion of AA and nHW participants exhibited at least one microbleed, though this was not formally tested for statistical significance.

Of exception, two studies reported significant differences between AA and nHW in ischemic lesion burden. In both of these studies, prevalence of infarcts in AA participants was greater than that of nHW participants (Wright et al., 2008; Qiao et al., 2016). Analyses from NOMAS found that among 656 participants, prevalence of at least one lesion was higher in AA participants (22%) compared to nHW participants (14%) (Wright et al., 2008). In a subsample of 1755 ARIC participants, investigators reported that 41% of AA participants and 32% of nHW participants exhibited at least one infarct (Qiao et al., 2016). Neither of these studies reported participant cognitive status.

In terms of infarct or lesion comparisons between Hispanics and nHW, no study reported statistically significant differences (DeCarli et al., 2008; Wiegman et al., 2014; Zahodne et al., 2015). One study from WHICAP that restricted to 638 participants with normal cognition or MCI, reported that Hispanics and nHW did not differ in the proportion of participants with infarcts (Zahodne et al., 2015). DeCarli et al. (2008) also found that subcortical infarcts did not vary significantly between nHW and Hispanics in a community- and ADRC-based study after adjusting for age, gender, education, and clinical diagnosis. In a another subset of WHICAP with 243 participants ranging from normal cognition to dementia, Wiegman et al. (2014) reported that relative to nHW, the proportion of Hispanics who had at least one microbleed did not significantly differ.

Studies included this review found that both AA and Hispanic race modified the relationship of cSVD on cognition such that minorities exhibited worse cognition than nHW at similar levels of burden. For example, one community- and ADRC-based study revealed an interaction between race and WMH volume such that WMH was associated with greater cognitive impairment in AA compared to nHW participants (Howell et al., 2017). Additionally, in a subset of CHS participants, Shadlen et al. (2006) found that infarcts were more strongly associated with dementia in AA participants compared to nHW. In contrast, analyses from CHAP revealed that the rate of cognitive decline with increasing WMH volume did not differ between nHW and AA (Aggarwal et al., 2010). However, nHW participants consistently displayed better global cognition compared to AA. Finally, one ADRC-based study found that cognitive impairment in Hispanics was worse at similar levels of visually-assessed WMH burden compared to nHW (Burke et al., 2018).

#### Sex Differences in cSVD Biomarkers by Race

Two studies assessed measurements of cSVD by sex and race. In a subsample of 702 WHICAP participants, among those with normal cognition and MCI, sex was not associated with WMH volume (Brickman et al., 2008). Additionally, there were no significant sex-by-race interactions for WMH. The other study that reported sex-by-race differences, a post-mortem analysis that did not report participants cognitive status, found varying sex differences by race as related to brain infarcts and lacunes (Riudavets et al., 2006). Among AA participants, 26% of women and 16% of men exhibited infarcts or lacunes. However, in nHW participants, 16% of women and 26% of men exhibited such lesions; this was not formally tested for statistical significance.

#### Summary and Synthesis

Before discussing results surrounding race-related differences in cSVD burden, it should be noted that the majority of comparisons for these biomarkers were conducted without covariate adjustment (Figure 2). Further, of those that did adjust for confounders, many did not control for cardiovascular risk factors (DeCarli et al., 2008; Burke et al., 2018; Rizvi et al., 2018). In addition to being risk factors for cSVD (Babulal et al., 2019), incidence and prevalence rates of cardiovascular diseases differ between races (Alzheimer’s Association, 2019) and sexes (Mozaffarian et al., 2015). Thus, some estimates of race and sex-by-race-related differences of cSVD may be biased due to unmeasured confounding.

Overall, findings for group differences in WMH between AA and nHW participants were mixed. Notably, the studies that reported no significant racial differences did not adjust for other covariates (Aggarwal et al., 2010; Gottesman et al., 2016; Howell et al., 2017; Amariglio et al., 2020). In contrast, those that reported relatively greater WMH in AA populations accounted for confounders in their analyses (Brickman et al., 2008; Della-Morte et al., 2018; Rizvi et al., 2018); these studies are further strengthened by having been conducted in large epidemiological cohorts. However, because they only represent two cohorts, both from Manhattan, New York, the findings may not generalize to other populations. Thus, the true magnitude of racial differences is likely not captured by these studies.

Results related to WMH burden in Hispanic populations consistently suggested that Hispanics exhibit lower WMH than nHW. While three studies found either no differences or greater burden in Hispanics, these findings may be influenced by lack of covariate adjustment (Zahodne et al., 2015) or, as the authors note in Minagar et al. (2000), the use of semiquantitative assessments with limited sensitivity.

In terms of comparisons between AA and nHW, only two studies that measured infarcts or lesions reported significant findings when comparing AA and nHW participants; in both studies, AA showed more frequent lesions and infarcts compared to nHW (Wright et al., 2008; Qiao et al., 2016). These findings are consistent with those that found greater WMH burden in AA populations relative to nHW and are further strengthened by having been uncovered in two large epidemiological studies, NOMAS and ARIC. In contrast, no studies that compared lesions or infarcts between Hispanics and nHW reported significant differences.

Previous work has found that female sex is associated with greater presence of lacunes and progression of WMH (van Dijk et al., 2008). This was not reflected in the WHICAP cohort, which found that women and men exhibited similar average volume of WMH (Brickman et al., 2008). In contrast, Riudavets et al. (2006) did report greater prevalence of infarcts and lacunes in women, but only in the AA populations. However, this comparison was not formally tested for significance, and thus, should be interpreted cautiously.

Several studies additionally reported a differential association of cSVD and cognition based on race; AA and Hispanic populations both exhibited worse cognitive function at similar burden of cSVD, indicating worse cognitive resilience to such pathology. These findings are particularly relevant for minority women as previous work has suggested that progression of WMH in women is faster than that in men (van Dijk et al., 2008). Thus, AA and Hispanic women may face higher risk of quickly-progressing cSVD coupled with relatively worse cognitive outcomes in its presence.

## Discussion

There is currently a limited number of studies that have examined ADRD pathology between races; there are even fewer that have done so by both race and sex. Despite this, we found evidence that greater prevalence of clinical ADRD among AA populations may be driven in part by more severe AD region-specific neurodegeneration and cSVD compared to other races. These findings were consistent in population-based studies among participants with varying cognitive status. However, we also found that both Hispanic populations and women show less severe pathology relative to nHW populations and men, respectively; both Hispanics and women exhibit relatively less neurodegeneration in regions affected by AD and cSVD. Thus, differences in neurodegeneration and cSVD on their own likely do not capture the full picture of sex-by-race differences in ADRD.

We did not find that minorities exhibited worse risk profiles for amyloid and tau pathology relative to nHW. Sex-by-race differences in Aß were additionally inconsistent. Although weak, there was some evidence for sex differences in tau severity such that a greater proportion of AA and nHW women exhibited more advanced tauopathy compared to men of their same race. This finding is consistent with results from largely nHW cohorts (Buckley et al., 2019; Ossenkoppele et al., 2020), implying that female sex may be associated with greater tau burden independent of race. Given that tau is neurodegenerative (Spillantini and Goedert, 2013), it follows that women should experience more atrophy in regions affected by such protein aggregation. However, as noted above, women generally show less AD-related neurodegeneration than men. Thus, it may be that independent of race, women exhibit better brain resilience than men in the face of tau accumulation.

Minorities also consistently showed worse cognition relative to nHW in the presence of comparable ADRD pathology, including Aß and cSVD. While the studies that reported these race-related differences did not additionally stratify by sex, previous work has found a similar pattern among women. At comparable levels of Aß deposition, women exhibit worse cognition relative to men (Gamberger et al., 2017; Koran et al., 2017). Others have found that among those with cSVD, women decline more quickly than men (van Dijk et al., 2008). Considering both lines of research, these findings suggest that minority women may be at especially high risk of cognitive decline in the presence of more than one neuropathological insult related to ADRD. It should be noted that while cognitive decline is highly correlated with cSVD in older adult populations (Prins and Scheltens, 2015), it is generally not strongly associated with Aß aggregation directly. Rather, severity of clinical AD symptomatology correlates more strongly with neurodegeneration caused by tau accumulation (Hedden et al., 2013; LaPoint et al., 2017; Jansen et al., 2018; Maass et al., 2018). Thus, it is possible that despite potentially exhibiting more tau and less neurodegeneration than men of their same race (e.g., more brain resilience), minority women are more cognitively vulnerable to neuropathological insult compared to both men and nHW populations (e.g., worse cognitive resilience), which may partially explain their relatively higher prevalence of ADRD.

There is a growing body of evidence that suggests pathological and clinical presentation of ADRD differ between men and women (Jack et al., 2015; Sundermann et al., 2016; Ferretti et al., 2018; Buckley et al., 2019; Ossenkoppele et al., 2020). Results of this review indicate that race may additionally alter these sex differences, potentially supporting emerging evidence of greater ADRD prevalence among AA and Hispanic women compared to other older adults (Matthews et al., 2019). Whether race functions additively or multiplicatively with sex on ADRD pathology, and further, downstream symptomatology, is currently unclear. This gap in knowledge is due to the small number of studies that have examined ADRD biomarkers by both race and sex. As such, sites with existing biomarker and cognitive outcomes in racially diverse cohorts should consider conducting additional analyses on their data by (1) testing sex-by-race interactions on pathology and cognition or (2) stratifying by both sex and race and making biomarker and cognition comparisons accordingly.

In terms of future data collection, investigators should aim to recruit more racially diverse cohorts from which ADRD biomarkers, cognition, and risk factors can be measured, and sex-by-race analyses can be conducted; relating biomarker and cognitive outcomes to risk factors may help identify differential ADRD mechanisms in minority women. Additionally, recruiting minority participants who are representative of the target population will increase the generalizability of study results. There is also a need for serial biomarker measurements in these populations as all studies included in this review were cross-sectional. As such, they provide limited insight into the natural history of ADRD in AA and Hispanic populations. Still, the data examined herein suggests potentially altered trajectories of ADRD progression in minorities and further, minority women. Finally, it should be noted that investigators have reported difficulty with recruiting minorities for neuroimaging and CSF studies (Morris et al., 2019; Amariglio et al., 2020). Thus, designing studies around emerging blood-based biomarkers, which are less invasive, may circumvent this obstacle, allowing for more generalizable samples and better retention rates in longitudinal studies. Such steps to better characterize ADRD pathology and its progression in minority women, especially in the context of the AT(N) framework, should inform better diagnostic and therapeutic techniques, ultimately benefiting those potentially most at risk.

In summary, through this review, we identified that women may exhibit more tau, but less neurodegeneration than men, independent of race. We additionally found that women are more likely to show relatively worse cognition at similar levels of pathology load. Thus, women of all races may have lower cognitive resilience to ADRD neuropathological insult compared to men, despite also possessing higher brain resilience. While race likely alters these sex differences, the specific mechanism and magnitude of this effect is currently unknown. Future studies should aim to fill this gap in knowledge by recruiting more diverse and representative cohorts, comparing ADRD biomarker severity by both race and sex, and collecting relevant risk factor and cognitive data.

## Author Contributions

SR and CR conceived and designed the review and drafted the manuscript. AC and BS reviewed and edited the manuscript. All authors contributed to the article and approved the submitted version.

## Conflict of Interest

The authors declare that the research was conducted in the absence of any commercial or financial relationships that could be construed as a potential conflict of interest.
