# Supplementary material for: Differences in Alzheimer’s Disease and Related Dementias Pathology Among African American and Hispanic Women: A Qualitative Literature Review of Biomarker Studies
Source: Front Syst Neurosci. 2021 Jul 21;15:685957. doi: 10.3389/fnsys.2021.685957 (PMC8334184; doi:10.3389/fnsys.2021.685957)
Supplement: Supplementary file 1 [file Table_1.docx]

Supplementary Material

**Supplementary Table 1.** Line-by-line search of OVID Medline

| **Line Number** | **Search String** |
| --- | --- |
| 1 | alzheimer disease/ or dementia/ or executive function/ or frontotemporal dementia/ or frontotemporal lobar degeneration/ |
| 2 | (Alzheimer* or “cognitive aging” or dementia or “executive function” or “executive functions” or “matter atrophy”).ti,ab,kw. |
| 3 | 1 or 2 |
| 4 | brain/ or broca area/ or ca1 region, hippocampal/ or ca2 region, hippocampal/ or ca3 region, hippocampal/ or cerebral cortex/ or dentate gyrus/ or entorhinal cortex/ or fornix, brain/ or frontal lobe/ or gray matter/ or gyrus cinguli/ or hippocampus/ or limbic lobe/ or mossy fibers, hippocampal/ or parahippocampal gyrus/ or periamygdaloid cortex/ or prefrontal cortex/ or white matter/ |
| 5 | (allocortex or amygdala or brain or brains or broca or ca1 or ca2 or ca3 or cerebral or cortex or diencephalon or epithalamus or forebrain or fornix or frontal lobe or frontal lobes or glymphatic system or gray matter or grey matter or gyrus or hippocampal or hippocampus or hypothalamus or insula or left hemisphere or limbic or meningeal artery or mossy fiber or mossy fibers or NAGM or NAWM or parahippocampal or periamygdaloid or pia artery or posterior cingulate or posterior communicating artery or posterior inferior cerebellar artery or right hemisphere or subcortex or subiculum or subthalamus or superior cerebellar artery or telencephalon or temporal lobe or thalamus or white matter).ti,ab,kw. |
| 6 | 4 or 5 |
| 7 | (atrophied or atrophy or size or volume or volumes).ti,ab,kw. Or Organ size/ |
| 8 | 6 and 7 |
| 9 | 3 or 8 |
| 10 | Cerebrospinal Fluid/ |
| 11 | (Cerebrospinal Fluid or Cerebrospinal Fluids or CSF or spinal fluid or spinals fluids).ti,ab,kw,rn. |
| 12 | cf.fs. |
| 13 | 10 or 11 or 12 |
| 14 | Amyloid beta-Peptides/ or Neurofilament Proteins/ or Peptide Fragments/ or Plaque, Amyloid/ or tau proteins/ |
| 15 | (amyloid or amyloids or filament or filaments or nfl or neurofilament or neurofilaments or peptide or peptides or plaque or plaques or tau).ti,ab,kw,rn. |
| 16 | 14 or 15 |
| 17 | 13 and 16 |
| 18 | 9 and 17 |
| 19 | cerebral angiography/ or cerebral ventriculography/ or computed tomography angiography/ or diffusion magnetic resonance imaging/ or diffusion tensor imaging/ or echoencephalography/ or echo-planar imaging/ or electron microscope tomography/ or fluorine-19 magnetic resonance imaging/ or magnetic resonance angiography/ or magnetic resonance imaging, cine/ or magnetic resonance imaging/ or multimodal imaging/ or myelography/ or neuroimaging/ or neuroradiography/ or pneumoencephalography/ or positron emission tomography computed tomography/ or positron-emission tomography/ or single photon emission computed tomography computed tomography/ or tau Proteins/ or tomography, emission-computed, single-photon/ or tomography, emission-computed/ or tomography/ or ultrasonography, doppler, transcranial/ or dg.fs. |
| 20 | (angiography or echoencephalography or fluorine-19 or imaging or mri or mris or myelography or neuroimaging or neuroradiograph* or neuroradiology or pet scan or pet scans or pneumoencephalography or tomography or ultrasonography or ventriculographies or ventriculography).ti,ab,kw. |
| 21 | dg.fs. |
| 22 | 19 or 20 or 21 |
| 23 | 9 and 22 |
| 24 | 18 or 23 |
| 25 | african americans/ or african continental ancestry group/ or ethnic groups/ or hispanic americans/ or mexican americans/ or minority groups/ or race factors/ |
| 26 | (African American* or africans or Black or Blacks or ethnic or ethnicities or ethnicity or ethnoracial or Hispanic or Hispanics or latino* or Mexican American* or Mexicans or minorities or minority or multiethnic or multiracial or race or racial).ti,ab,kw. |
| 27 | 25 or 26 |
| 28 | 24 and 27 |
| 29 | women/ or female/ |
| 30 | (female* or women).ti,ab,kw. |
| 31 | Sex Factors/ |
| 32 | (sex adj3 (differences or factors)).ti,ab,kw. |
| 33 | 29 or 30 or 31 or 32 |
| 34 | 28 and 33 |
| 35 | 34 and english.la. |
| 36 | exp africa/ or exp asia/ or exp australia/ or exp canada/ or exp central america/ or exp europe/ or exp south america/ or “Caribbean Region”/ or Aruba/ or “Caribbean Netherlands”/ or Curacao/ or “Sint Maarten”/ or “West Indies”/ or “Antigua and Barbuda”/ or Bahamas/ or Barbados/ or “British Virgin Islands”/ or Cuba/ or Dominica/ or “Dominican Republic”/ or Grenada/ or Guadeloupe/ or Haiti/ or Jamaica/ or Martinique/ or “Saint Kitts and Nevis”/ or Saint Lucia/ or “Saint Vincent and the Grenadines”/ or “Trinidad and Tobago”/ |
| 37 | 35 not (36 not (36 and (north america/ or exp united states/))) |
