# Supplementary material for: Differences in Alzheimer’s Disease and Related Dementias Pathology Among African American and Hispanic Women: A Qualitative Literature Review of Biomarker Studies
Source: Front Syst Neurosci. 2021 Jul 21;15:685957. doi: 10.3389/fnsys.2021.685957 (PMC8334184; doi:10.3389/fnsys.2021.685957)
Supplement: Supplementary file 2 [file Table_2.DOCX]

Supplementary Material

**Supplementary Table 2.** Summary of literature included in the review, outlined according to use of biomarker (amyloid, tau, neurodegeneration, or cSVD).

|  | ***Reference*** | ***Recruited?*** | ***Race*** | ***N*** | ***Age*** | ***Women*** | ***APOE-ε4*** | ***Diagnosis*** | ***Covariates*** | ***Biomarker*** | ***Race differences*** | ***Sex-by-race differences*** |
| --- | --- | --- | --- | --- | --- | --- | --- | --- | --- | --- | --- | --- |
| **Amyloid** | *Amariglio et al. (2020)* | HABS: Boston, MA | nHW | 249 | 72.6 ± 7.2 | 79% | 30% | CN | - | PiB | nHW = AA | - |
|  |  |  | AA | 48 | 72.0 ± 6.3 | 59% | 26% |  |  |  |  |  |
|  | *Duara et al. (2019)* | ADRC: Miami, FL | nHW | 65 | 70.3 ± 6.1 | 66% | 33% | CN, MCI, dementia | Age, MMSE, APOE | FBB | nHW = Hispanics | - |
|  |  |  | Hispanic | 94 |  |  |  |  |  |  |  |  |
|  | *Garrett et al. (2019)* | ADRC and community: Atlanta, GA | nHW | 210 | 65.6 ± 7.9 | 131 | - | CN, MCI | Age, sex, education, family history of AD, BMI, MoCA, HTN, diabetes, income | Aß-42 | nHW = AA | - |
|  |  |  | AA | 152 |  | 99 |  |  |  |  |  |  |
|  | *Gottesman et al. (2016)* | ARIC: Washington County, MD; Forsyth County, NC; Jackson, MS | nHW | 188 | 76.1 ± 5.6 | 53% | 26.6% | Dementia-free | Age, sex, race, education, APOE, HTN, diabetes, cognitive status, WMH volume | FBP | nHW < AA | w > m |
|  |  |  | AA | 141 | 75.5 ± 5.1 | 62% | 37.2% |  |  |  |  |  |
|  | *Gu et al. (2015)* | WHICAP: Northern Manhattan, NYC, NY | nHW | 40 | 84.5 ± 4.6 | 35% | 31% | Dementia-free | Age, sex, race, education, APOE | FBB | nHW = AA = Hispanics | - |
|  |  |  | AA | 53 |  | 46% |  |  |  |  |  |  |
|  |  |  | Hispanic | 22 |  | 19% |  |  |  |  |  |  |
|  | *Han et al. (2020)* | ADRC: Davis, CA | nHW | 83 | 74.2 ± 6.9 | 50% | - | CN | - | FBP | Hispanic > nHW > AA | - |
|  |  |  | AA | 23 |  |  |  |  |  |  |  |  |
|  |  |  | Hispanic | 34 |  |  |  |  |  |  |  |  |
|  | *Howell et al. (2017)* | ADRC and community: Atlanta, GA | nHW | 70 | 70.8 ± 7.7 | 59% | 50% | CN, MCI, Dementia | - | Aß-42 | nHW = AA | - |
|  |  |  | AA | 65 | 69.1 ± 7.4 | 55% | 52% |  |  |  |  |  |
|  | *Morris et al. (2019)* | Community: St. Louis, MO | nHW | 1082 | 70.8 ± 9.9 | 55% | 42% | CN, MCI, AD | Sex, APOE, age, education, clinical status, family history of AD, BMI, CSF drift variables | 1) PiB  2) Aß-42 | 1, 2) nHW = AA | - |
|  |  |  | AA | 173 | 70.8 ± 9.6 | 65% | 46% |  |  |  |  |  |
|  | *Riudavets et al. (2006)* | Community: MD | nHW | 100 | w = 80 ± 8.3  m = 76.1 ± 7.8 | 50 | w = 26%, m =18% | - | Age | Post-mortem tissue | nHW = AA | w >/= m |
|  |  |  | AA | 100 | w = 75 ± 7.6  m = 73 ± 7.3 | 50 | w = 30%  m = 44% |  |  |  |  |  |
| **Tau** | *Garrett et al. (2019)* | ADRC and community: Atlanta, GA | nHW | 210 | 65.6 ± 7.9 | 131 | - | CN, MCI | Age, sex, education, family history of AD, body mass index, MoCA, HTN, diabetes, and income | 1) t-tau  2) p-tau | 1,2)  MCI:  nHW > AA  CN:  nHW = AA | - |
|  |  |  | AA | 152 |  | 99 |  |  |  |  |  |  |
|  | *Howell et al. (2017)* | ADRC and community: Atlanta, GA | nHW | 70 | 70.8 ± 7.7 | 59% | 50% | CN, MCI, Dementia | Cognitive function, age, sex, APOE, ABCA7, Aß-42 | 1) t-tau  2) p-tau | 1, 2) nHW > AA | - |
|  |  |  | AA | 65 | 69.1 ± 7.4 | 55% | 52% |  |  |  |  |  |
|  | *Lee et al. (2018)* | HABS: Boston, MA | nHW | 123 | 76 | 54% | - | - | Age, sex, education, Aß | FTP | nHW < AA | - |
|  |  |  | AA | 23 | 73 | 83% |  |  |  |  |  |  |
|  | *Morris et al. (2019)* | Community: St. Louis, MO | nHW | 1082 | 70.8 ± 9.9 | 55% | 41.7% | CN, MCI, AD | Sex, APOE, age, education, clinical status, family history of AD, BMI, CSF drift variables | 1) t-tau  2) p-tau | 1,2) nHW > AA | - |
|  |  |  | AA | 173 | 70.8 ± 9.6 | 65% | 45.6% |  |  |  |  |  |
|  | *Rajan et al. (2020)* | CHAP: Chicago, IL | nHW | 516 | 75.4 ± 6.8 | 61% | 29% | CN | - | 1) t-tau | nHW = AA | - |
|  |  |  | AA | 811 | 72.2 ± 5.9 | 61% | 36% |  |  |  |  |  |
|  | *Riudavets et al. (2006)* | Community: MD | nHW | 100 | w = 80 ± 8.3  m = 76.1 ± 7.8 | 50 | w = 26%, m =18% | - | Age | Post-mortem tissue | nHW = AA | w > m |
|  |  |  | AA | 100 | w = 75 ± 7.6  m = 73 ± 7.3 | 50 | w = 30%  m = 44% |  |  |  |  |  |
| **Neurodegeneration** | *Aggarwal et al. (2010)* | CHAP: Chicago, IL | nHW | 240 | 80.1 ± 5.8 | 58% | - | CN, MCI, dementia | - | Global volume | nHW = AA | - |
|  |  |  | AA | 335 | 79.5 ± 6.0 | 57% |  |  |  |  |  |  |
|  | *Arruda et al. (2020)* | ADRC: Miami, FL | nHW | 89 | 72.8 ± 8.3 | 57% | - | CN, MCI, AD | Age, education, ethnicity, gender, MoCA, FAQ | 1) HC volume  2) EC volume  3) EC thickness | 1, 2) nHW < Hispanic  3) nHW = Hispanic | 1) w > m  2) w = m  3) w = m |
|  |  |  | Hispanic | 137 | 71.1 ± 7.6 | 63% |  |  |  |  |  |  |
|  | *Brickman et al. (2008)* | WHICAP: Northern Manhattan, NYC, NY | nHW | 203 | 80.3 ± 5.7 | 121 | - | CN, MCI | Age, sex, vascular disease | 1) Global volume  2) HC volume  3) EC volume  4) Ventricle size | 1) nHW < AA, Hispanic  2, 3) nHW = AA = Hispanic  4) nHW > AA, Hispanic | 1, 4) w < m  2, 3) w = m |
|  |  |  | AA | 243 | 79.7 ± 5.8 | 169 |  |  |  |  |  |  |
|  |  |  | Hispanic | 256 | 80.3 ± 5.2 | 189 |  |  |  |  |  |  |
|  | *Burke et al. (2018)* | ADRC: Miami, FL | nHW | 165 | ~77 | ~55% | - | CN, MCI, Dementia | Age, education, Geriatric Depression Scale score | 1) HC volume  2) EC volume  3) Ventricle volume | 1, 2, 3) nHW < Hispanic | - |
|  |  |  | Hispanic |  | ~74 | ~64% |  |  |  |  |  |  |
|  | *DeCarli et al. (2008)* | ADRC and community: Davis, CA | nHW | 191 | 75.3 ± 7.5 | 109 | - | CN, MCI, dementia | Age, sex, education, cognitive status | 1) Global volume  2) HC volume | 1) nHW = AA = Hispanic  2)  CN, dementia: nHW > AA, Hispanic  MCI:  nHW < AA, Hispanic | - |
|  |  |  | AA | 103 | 74.5 ± 6.9 | 69 |  |  |  |  |  |  |
|  |  |  | Hispanic | 107 | 72.6 ± 7.4 | 74 |  |  |  |  |  |  |
|  | *Duara et al. (2019)* | ADRC: Miami, FL | nHW | 159 | 70.3 ± 6.1 | 66% | 33% | CN, MCI, dementia | - | HC volume | nHW = Hispanic | - |
|  |  |  | Hispanic |  |  |  |  |  |  |  |  |  |
|  | *Garrett et al. (2019)* | ADRC and community: Atlanta, GA | nHW | 210 | 65.6 ± 7.9 | 131 | - | CN, MCI | Age, sex, education, family history of AD, body mass index, MoCA, hypertension, diabetes, and income | HC volume | nHW = AA | - |
|  |  |  | AA | 152 |  | 99 |  |  |  |  |  |  |
|  | *Howell et al. (2017)* | ADRC and community: Atlanta, GA | nHW | 70 | 70.8 ± 7.7 | 59% | 50% | CN, MCI, Dementia | - | 1) HC volume  2) NfL | 1) nHW = AA  2)  *CN*: nHW > AA;  *MCI + dementia:* nHW = AA | - |
|  |  |  | AA | 65 | 69.1 ± 7.4 | 55% | 52% |  |  |  |  |  |
|  | *Minagar et al. (2000)* | - | nHW | 73 | 76 | 66% | - | AD | Age, education, gender, MMSE, disease duration | 1) Cortical atrophy  2) Ventricle size | 1) nHW = Hispanic  2) nHW > Hispanic | - |
|  |  |  | Hispanic | 71 | 74 | 71% |  |  |  |  |  |  |
|  | *McDonough (2017)* | HABS: Boston, MA | nHW | 232 | ~72 | 30 | - | - | Propensity scores created using: age, sex, education, verbal IQ, AB level, WM hypointensity | AD-signature cortical thickness | nHW > AA | - |
|  |  |  | AA | 43 | ~73 | 32 |  |  |  |  |  |  |
|  | *Morris et al. (2019)* | Community: St. Louis, MO | nHW | 1082 | 70.8 ± 9.9 | 55% | 41.7% | CN, MCI, AD | Sex, APOE e4 status, age, education level, clinical status (CDR < or > 0), family history of AD | HC volume | nHW > AA | - |
|  |  |  | AA | 173 | 70.8 ± 9.6 | 65% | 45.6% |  |  |  |  |  |
|  | *Rizvi et al. (2018)* | WHICAP: Northern Manhattan, NYC, NY | nHW | 173 | 73.7 ± 5.6 | 50% | - | CN, MCI, dementia | Age, education, intracranial volume | Global cortical thickness | nHW > AA, Hispanic | - |
|  |  |  | AA | 188 | 74.1 ± 6.0 | 63% |  |  |  |  |  |  |
|  |  |  | Hispanic | 158 | 74.1 ± 5.3 | 54% |  |  |  |  |  |  |
|  | *Rajan et al. (2020)* | CHAP: Chicago, IL | nHW | 516 | 75.4 ± 6.8 | 61% | 29% | CN | - | NfL | nHW > AA |  |
|  |  |  | AA | 811 | 72.2 ± 5.9 | 61% | 36% |  |  |  |  |  |
|  | *Shadlen et al. (2006)* | CHS: Forsyth County, NC, Sacramento County, CA, Washington County, MD, Pittsburgh, PA | nHW | 2503 | - | 1458 | - | Dementia-free, dementia | - | Ventricle size | nHW > AA | - |
|  |  |  | AA | 283 |  | 183 |  |  |  |  |  |  |
|  | *Zahodne et al. (2015)* | WHICAP: Northern Manhattan, NYC, NY | nHW | 184 | 80.2 ± 5.6 | 59% | - | CN, MCI | - | 1) HC volume  2) AD- signature cortical thickness | 1) nHW > AA, Hispanic  2) Hispanic = nHW > AA | - |
|  |  |  | AA | 229 | 79.8 ± 5.7 | 69% |  |  |  |  |  |  |
|  |  |  | Hispanic | 225 | 80.2 ± 5.2 | 74% |  |  |  |  |  |  |
| **cSVD** | *Aggarwal et al. (2010)* | CHAP: Chicago, IL | nHW | 240 | 80 | 58% | - | CN, MCI, dementia | - | 1) WMH  2) Infarcts | 1, 2) nHW = AA | - |
|  |  |  | AA | 335 | 79 | 57% |  |  |  |  |  |  |
|  | *Amariglio et al. (2020)* | HABS: Boston, MA | nHW | 248 | 72.6 ± 7.2 |  | - | CN | - | WMH | nHW = AA | - |
|  |  |  | AA | 48 | 72.0 ± 6.3 | 48% |  |  |  |  |  |  |
|  | *Brickman et al. (2008)* | WHICAP: Northern Manhattan, NYC, NY | nHW | 203 | 80.3 ± 5.7 | 121 | - | CN, MCI | Age, sex, vascular disease | WMH | nHW < AA, Hispanic | w = m |
|  |  |  | AA | 243 | 79.7 ± 5.8 | 169 |  |  |  |  |  |  |
|  |  |  | Hispanic | 256 | 80.3 ± 5.2 | 189 |  |  |  |  |  |  |
|  | *Burke et al. (2018)* | ADRC: Miami, FL | nHW | 165 | ~77 | ~55% | - | CN, MCI, Dementia | Age, education, geriatric depression scale scores | WMH | nHW > Hispanic | - |
|  |  |  | Hispanic |  | ~74 | ~64% |  |  |  |  |  |  |
|  | *DeCarli et al. (2008)* | ADRC and community: Davis, CA | nHW | 191 | 75.3 ± 7.5 | 109 | - | CN, MCI, dementia | Age, gender, education, cognitive status | 1) WMH  2) Infarcts | 1, 2) nHW = AA, Hispanic | 1, 2) w = m |
|  |  |  | AA | 103 | 74.5 ± 6.9 | 69 |  |  |  |  |  |  |
|  |  |  | Hispanic | 107 | 72.6 ± 7.4 | 74 |  |  |  |  |  |  |
|  | *Della-Morte et al. (2018)* | NOMAS: Northern Manhattan, NYC, NY | nHW | 1229 | 71 ± 9 | 60% | - | - | - | WMH | Hispanic > nHW > AA | - |
|  |  |  | AA |  |  |  |  |  |  |  |  |  |
|  |  |  | Hispanic |  |  |  |  |  |  |  |  |  |
|  | *Gottesman et al. (2016)* | ARIC: Washington County, MD; Forsyth County, NC; Jackson, MS | 188 | 76.1 ± 5.6 | 53% | 26.6% | - | Dementia free | - | WMH | nHW = AA | - |
|  |  |  | 141 | 75.5 ± 5.1 | 62% | 37.2% |  |  |  |  |  |  |
|  | *Howell et al. (2017)* | ADRC and community: Atlanta, GA | nHW | 70 | 70.8 ± 7.7 | 59% | 50% | CN, MCI, Dementia | - | WMH | nHW = AA | - |
|  |  |  | AA | 65 | 69.1 ± 7.4 | 55% | 52% |  |  |  |  |  |
|  | *Minagar et al. (2000)* | - | nHW | 73 | 76 | 66% | - | AD | Age, education, gender, MMSE, AD duration | WMH | nHW = Hispanic | - |
|  |  |  | Hispanic | 71 | 74 | 71% |  |  |  |  |  |  |
|  | *Morris et al. (2019)* | Community: St. Louis, MO | nHW | 1082 | 70.8 ± 9.9 | 55% | 41.7% | CN, MCI, AD | - | Ischemic lesions | nHW = AA | - |
|  |  |  | AA | 173 | 70.8 ± 9.6 | 65% | 45.6% |  |  |  |  |  |
|  | *Qiao et al. (2016)* | ARIC: Washington County, MD; Forsyth County, NC; Jackson, MS | nHW | 1234 | 77.6 | 58% | - | - | - | Intracranial lesions | nHW < AA | - |
|  |  |  | AA | 518 |  |  |  |  |  |  |  |  |
|  | *Rizvi et al. (2018)* | WHICAP: Northern Manhattan, NYC, NY | nHW | 173 | 73.7 ± 5.6 | 50% | - | CN, MCI, dementia | Age, education, intracranial volume | WMH | Hispanic < nHW < AA | - |
|  |  |  | AA | 188 | 74.1 ± 6.0 | 63% |  |  |  |  |  |  |
|  |  |  | Hispanic | 158 | 74.1 ± 5.3 | 54% |  |  |  |  |  |  |
|  | *Riudavets et al. (2006)* | Community: MD | nHW | 100 | w = 80 ± 8.3  m = 76.1 ± 7.8 | 50 | w = 26%, m =18% | - | Age | Infarcts | - | *AA*: w > m  *nHW:* w < m |
|  |  |  | AA | 100 | w = 75 ± 7.6  m = 73 ± 7.3 | 50 | w = 30%  m = 44% |  |  |  |  |  |
|  | *Shadlen et al. (2006)* | CHS: Forsyth County, NC, Sacramento County, CA, Washington County, MD, Pittsburgh, PA | nHW | 2503 |  | 1458 | - | Dementia-free, dementia | - | WMH | nHW = AA | - |
|  |  |  | AA | 283 |  | 183 |  |  |  |  |  |  |
|  | *Wiegman et al. (2014)* | WHICAP: Northern Manhattan, NYC, NY | nHW | 243 | 84 | 71% | - | CN, MCI, dementia | - | Microbleeds | nHW = Hispanic < AA | - |
|  |  |  | AA |  |  |  |  |  |  |  |  |  |
|  |  |  | Hispanic |  |  |  |  |  |  |  |  |  |
|  | *Wright et al. (2008)* | NOMAS: Northern Manhattan | nHW | 18% | 70.4 ± 7.9 | 59% | - | - | - | Infarcts | Hispanic = nHW < AA | - |
|  |  |  | AA | 21% |  |  |  |  |  |  |  |  |
|  |  |  | Hispanic | 61% |  |  |  |  |  |  |  |  |
|  | *Zahodne et al. (2015)* | WHICAP: Northern Manhattan, NYC, NY | nHW | 184 | 80.2 ± 5.6 | 59% | - | CN, MCI | - | 1) WMH  2) Infarcts | 1) nHW < AA, Hispanic  2) nHW = AA, Hispanic | - |

HABS (Harvard Aging Brain Study); nHW (non-Hispanic white); AA (African American); CN (cognitively normal); PiB (Pittsburgh Compound-B); ADRC (Alzheimer’s disease Research Center); MCI (Mild Cognitive Impairment); MMSE (Mini Mental State Examination); FBB (Florbetaben); MoCA (Montreal Cognitive Assessment); BMI (body mass index); HTN (hypertension); ARIC (Atherosclerosis Risk in Communities study); FBP (Florbetapir); WMH (white matter hyperintensities); WHICAP (Washington Heights-Inwood Community Aging Project); CHAP (Chicago Healthy Aging Project); FAQ (Functional Activities Questionnaire); HC (hippocampus); EC (entorhinal cortex); CHS (Community Health Study); NOMAS (Northern Manhattan Study)

Bibliography

Aggarwal, N. T., Wilson, R. S., Bienias, J. L., De Jager, P. L., Bennett, D. A., Evans, D. A., and DeCarli, C. (2010). The association of magnetic resonance imaging measures with cognitive function in a biracial population sample. *Arch. Neurol.* 67, 475–482. doi:10.1001/archneurol.2010.42.

Amariglio, R. E., Buckley, R. F., Rabin, J. S., Papp, K. V., Quiroz, Y. T., Mormino, E. C., Sparks, K. P., Johnson, K. A., Rentz, D. M., and Sperling, R. A. (2020). Examining cognitive decline across black and white participants in the harvard aging brain study. *J. Alzheimers Dis.* 75, 1437–1446. doi:10.3233/JAD-191291.

Arruda, F., Rosselli, M., Greig, M. T., Loewenstein, D. A., Lang, M., Torres, V. L., Vélez-Uribe, I., Conniff, J., Barker, W. W., Curiel, R. E., et al. (2020). The association between functional assessment and structural brain biomarkers in an ethnically diverse sample with normal cognition, mild cognitive impairment, or dementia. *Arch Clin Neuropsychol*. doi:10.1093/arclin/acaa065.

Brickman, A. M., Schupf, N., Manly, J. J., Luchsinger, J. A., Andrews, H., Tang, M. X., Reitz, C., Small, S. A., Mayeux, R., DeCarli, C., et al. (2008). Brain morphology in older African Americans, Caribbean Hispanics, and whites from northern Manhattan. *Arch. Neurol.* 65, 1053–1061. doi:10.1001/archneur.65.8.1053.

Burke, S. L., Rodriguez, M. J., Barker, W., Greig-Custo, M. T., Rosselli, M., Loewenstein, D. A., and Duara, R. (2018). Relationship between Cognitive Performance and Measures of Neurodegeneration among Hispanic and White Non-Hispanic Individuals with Normal Cognition, Mild Cognitive Impairment, and Dementia. *J. Int. Neuropsychol. Soc.* 24, 176–187. doi:10.1017/S1355617717000820.

DeCarli, C., Reed, B. R., Jagust, W., Martinez, O., Ortega, M., and Mungas, D. (2008). Brain behavior relationships among African Americans, whites, and Hispanics. *Alzheimer Dis Assoc Disord* 22, 382–391. doi:10.1097/wad.0b013e318185e7fe.

Della-Morte, D., Dong, C., Markert, M. S., Elkind, M. S. V., Sacco, R. L., Wright, C. B., and Rundek, T. (2018). Carotid Intima-Media Thickness Is Associated With White Matter Hyperintensities: The Northern Manhattan Study. *Stroke* 49, 304–311. doi:10.1161/STROKEAHA.117.018943.

Duara, R., Loewenstein, D. A., Lizarraga, G., Adjouadi, M., Barker, W. W., Greig-Custo, M. T., Rosselli, M., Penate, A., Shea, Y. F., Behar, R., et al. (2019). Effect of age, ethnicity, sex, cognitive status and APOE genotype on amyloid load and the threshold for amyloid positivity. *Neuroimage Clin.* 22, 101800. doi:10.1016/j.nicl.2019.101800.

Garrett, S. L., McDaniel, D., Obideen, M., Trammell, A. R., Shaw, L. M., Goldstein, F. C., and Hajjar, I. (2019). Racial disparity in cerebrospinal fluid amyloid and tau biomarkers and associated cutoffs for mild cognitive impairment. *JAMA Netw. Open* 2, e1917363. doi:10.1001/jamanetworkopen.2019.17363.

Gottesman, R. F., Schneider, A. L. C., Zhou, Y., Chen, X., Green, E., Gupta, N., Knopman, D. S., Mintz, A., Rahmim, A., Sharrett, A. R., et al. (2016). The ARIC-PET amyloid imaging study: Brain amyloid differences by age, race, sex, and APOE. *Neurology* 87, 473–480. doi:10.1212/WNL.0000000000002914.

Gu, Y., Razlighi, Q. R., Zahodne, L. B., Janicki, S. C., Ichise, M., Manly, J. J., Devanand, D. P., Brickman, A. M., Schupf, N., Mayeux, R., et al. (2015). Brain Amyloid Deposition and Longitudinal Cognitive Decline in Nondemented Older Subjects: Results from a Multi-Ethnic Population. *PLoS One* 10, e0123743. doi:10.1371/journal.pone.0123743.

Han, J. W., Maillard, P., Harvey, D., Fletcher, E., Martinez, O., Johnson, D. K., Olichney, J. M., Farias, S. T., Villeneuve, S., Jagust, W., et al. (2020). Association of vascular brain injury, neurodegeneration, amyloid and cognitive trajectory. *Neurology*. doi:10.1212/WNL.0000000000010531.

Howell, J. C., Watts, K. D., Parker, M. W., Wu, J., Kollhoff, A., Wingo, T. S., Dorbin, C. D., Qiu, D., and Hu, W. T. (2017). Race modifies the relationship between cognition and Alzheimer’s disease cerebrospinal fluid biomarkers. *Alzheimers Res. Ther.* 9, 88. doi:10.1186/s13195-017-0315-1.

Lee, C. M., Jacobs, H. I. L., Marquié, M., Becker, J. A., Andrea, N. V., Jin, D. S., Schultz, A. P., Frosch, M. P., Gómez-Isla, T., Sperling, R. A., et al. (2018). 18F-Flortaucipir Binding in Choroid Plexus: Related to Race and Hippocampus Signal. *J. Alzheimers Dis.* 62, 1691–1702. doi:10.3233/JAD-170840.

McDonough, I. M. (2017). Beta-amyloid and Cortical Thickness Reveal Racial Disparities in Preclinical Alzheimer’s Disease. *NeuroImage: Clinical* 16, 659–667. doi:10.1016/j.nicl.2017.09.014.

Minagar, A., Sevush, S., and Bertran, A. (2000). Cerebral ventricles are smaller in Hispanic than non-Hispanic patients with Alzheimer’s disease. *Neurology* 55, 446–448. doi:10.1212/wnl.55.3.446.

Morris, J. C., Schindler, S. E., McCue, L. M., Moulder, K. L., Benzinger, T. L. S., Cruchaga, C., Fagan, A. M., Grant, E., Gordon, B. A., Holtzman, D. M., et al. (2019). Assessment of racial disparities in biomarkers for alzheimer disease. *JAMA Neurol.* 76, 264–273. doi:10.1001/jamaneurol.2018.4249.

Rajan, K. B., Aggarwal, N. T., McAninch, E. A., Weuve, J., Barnes, L. L., Wilson, R. S., DeCarli, C., and Evans, D. A. (2020). Remote blood biomarkers of longitudinal cognitive outcomes in a population study. *Ann. Neurol.* doi:10.1002/ana.25874.

Riudavets, M. A., Rubio, A., Cox, C., Rudow, G., Fowler, D., and Troncoso, J. C. (2006). The prevalence of Alzheimer neuropathologic lesions is similar in blacks and whites. *J. Neuropathol. Exp. Neurol.* 65, 1143–1148. doi:10.1097/01.jnen.0000248548.20799.a3.

Rizvi, B., Narkhede, A., Last, B. S., Budge, M., Tosto, G., Manly, J. J., Schupf, N., Mayeux, R., and Brickman, A. M. (2018). The effect of white matter hyperintensities on cognition is mediated by cortical atrophy. *Neurobiol. Aging* 64, 25–32. doi:10.1016/j.neurobiolaging.2017.12.006.

Shadlen, M.-F., Siscovick, D., Fitzpatrick, A. L., Dulberg, C., Kuller, L. H., and Jackson, S. (2006). Education, cognitive test scores, and black-white differences in dementia risk. *J. Am. Geriatr. Soc.* 54, 898–905. doi:10.1111/j.1532-5415.2006.00747.x.

Wiegman, A. F., Meier, I. B., Schupf, N., Manly, J. J., Guzman, V. A., Narkhede, A., Stern, Y., Martinez-Ramirez, S., Viswanathan, A., Luchsinger, J. A., et al. (2014). Cerebral microbleeds in a multiethnic elderly community: demographic and clinical correlates. *J. Neurol. Sci.* 345, 125–130. doi:10.1016/j.jns.2014.07.024.

Wright, C. B., Festa, J. R., Paik, M. C., Schmiedigen, A., Brown, T. R., Yoshita, M., DeCarli, C., Sacco, R., and Stern, Y. (2008). White matter hyperintensities and subclinical infarction: associations with psychomotor speed and cognitive flexibility. *Stroke* 39, 800–805. doi:10.1161/STROKEAHA.107.484147.

Zahodne, L. B., Manly, J. J., Narkhede, A., Griffith, E. Y., DeCarli, C., Schupf, N. S., Mayeux, R., and Brickman, A. M. (2015). Structural MRI Predictors of Late-Life Cognition Differ Across African Americans, Hispanics, and Whites. *Curr Alzheimer Res* 12, 632–639. doi:10.2174/1567205012666150530203214.
